# The Toxic Effects of Hydrated Cement, Autoclaved Aerated Concrete, and Demolition Dusts on the Respiratory System in Rats

**DOI:** 10.3390/toxics14030218

**Published:** 2026-03-03

**Authors:** Murat Kilic, Nurcan Gokturk, Nigar Vardi, Onural Ozhan, Gokce Koca, Mehmet Akif Turkoz, Merve Biyikli, Paki Turgut, Yusuf Turkoz, Hakan Parlakpinar, Eylem Karadag, Cemil Colak

**Affiliations:** 1 Department of Thoracic Surgery, Faculty of Medicine, Inonu University, 44280 Malatya, Türkiye; merve.biyikli@inonu.edu.tr; 2 Department of Medical Biochemistry, Faculty of Medicine, Inonu University, 44280 Malatya, Türkiye; 38193834001@ogr.inonu.edu.tr (N.G.); yusuf.turkoz@inonu.edu.tr (Y.T.); 3 Department of Histology and Embryology, Faculty of Medicine, Inonu University, 44280 Malatya, Türkiye; nigar.vardi@inonu.edu.tr (N.V.); gokce.koca@inonu.edu.tr (G.K.); 4 Department of Medical Pharmacology, Faculty of Medicine, Inonu University, 44280 Malatya, Türkiye; onural.ozhan@inonu.edu.tr (O.O.); hakan.parlakpinar@inonu.edu.tr (H.P.); 38244201004@ogr.inonu.edu.tr (E.K.); 5 Department of Radiology, Faculty of Medicine, Inonu University, 44280 Malatya, Türkiye; mehmet.turkoz@inonu.edu.tr; 6 Department of Civil Engineering, Inonu University, 44280 Malatya, Türkiye; paki.turgut@inonu.edu.tr; 7 Department of Biostatistics and Medical Informatics, Faculty of Medicine, Inonu University, 44280 Malatya, Türkiye; cemil.colak@inonu.edu.tr

**Keywords:** hydrated cement dust, autoclaved aerated concrete dust, demolition dust, earthquake, air pollution exposure

## Abstract

Background: Following the earthquakes that occurred in Türkiye in 2023, the resulting demolition dust (DD) negatively impacted air quality and led to an increase in respiratory diseases. Although the harmful effects of crystalline and amorphous silica are known, the effects of hydrated cement dust (HCD), autoclaved aerated concrete dust (AACD), and DD on the lungs have not been sufficiently investigated. This rat study presents the first experimental data on the toxicity of these dusts. Methods: In the study, the structural properties of dust particles smaller than 5 µm were characterized using XRD analysis. Subsequently, 48 female rats were divided into four groups: HCD, AACD, DD, and control. The relevant dust suspensions were administered to the experimental groups, and physiological saline was administered to the control group intranasally a total of five times over a 15-day period, once every 3 days. Subsequently, bronchoalveolar lavage fluid, blood, and lung tissues were analyzed. Results: An increase in emphysema was observed in all exposure groups, and this increase was significant in the AAC and HC groups. Inflammation and alveolar wall thickness increased in the HC and DD groups. Goblet cell hyperplasia was detected only in the HC group; increases in CD68^+^ macrophages and TGF-β, as well as elevated hydroxyproline, were detected only in the DD group and supported the fibrotic response (*p* < 0.05). Neutrophil increase was specific to the AAC group. In all exposure groups, Akt/NF-κB pathway proteins, caspase-9, and MPO levels increased, while Bcl-xl levels decreased (*p* < 0.05). The findings indicate that the examined dusts trigger inflammation and apoptosis. Conclusion: Exposure to HCD, AACD, and DD causes lung damage by modulating the Akt/NF-κB signaling cascade; it enhances the apoptotic process through Bcl-xl suppression and caspase-9 increase. DD also induces a marked fibrotic response.

## 1. Introduction

Airborne particulate matter is a well-established environmental risk factor for respiratory morbidity, as fine and ultrafine particles can penetrate deep into the lung parenchyma, inducing inflammation, oxidative stress, and long-term structural damage. Prolonged exposure to mineral- and construction-derived dusts has been associated with an increased incidence of respiratory diseases, including chronic bronchitis, pulmonary fibrosis, and impaired lung function [1].

**Figure 1:**
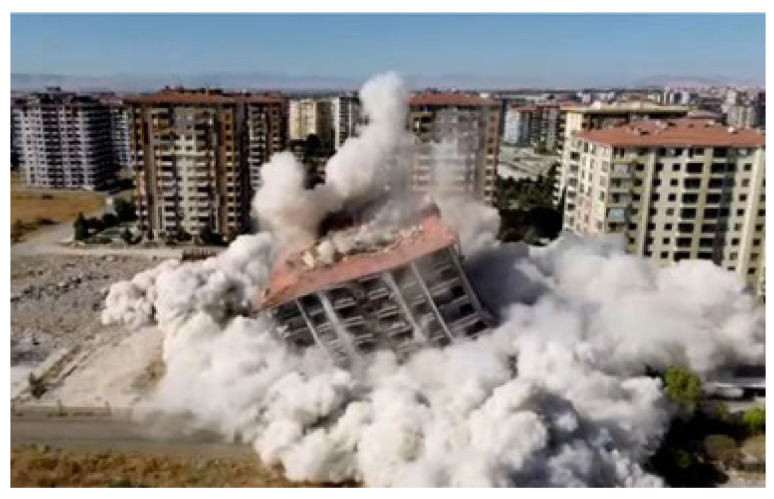
Dust generated during building demolition.

Following major earthquakes, air quality often deteriorates substantially due to large-scale structural damage and prolonged post-disaster activities. In this context, a devastating earthquake sequence occurred in Türkiye in 2023, affecting a vast geographical region and millions of people. Contrary to the common assumption that earthquake-related dust exposure is short-term, field observations indicate that airborne dust levels remain elevated for months or even years. Debris removal operations, the controlled demolition of severely damaged buildings using heavy machinery or explosives, the transportation of demolition dust (DD), and the long-term storage of rubble in open disposal areas collectively contribute to continuous emissions of particulate matter. These dusts primarily originate from building materials such as hydrated cement (HCD), autoclaved aerated concrete (AACD), and DD components commonly used in urban structures (Figure 1).

In regions experiencing extensive destruction, the duration and intensity of dust exposure are closely linked to the pace of debris removal and reconstruction activities. Severe deterioration in air quality has been documented in heavily affected provinces. For example, air quality monitoring data from Malatya province in 2023 demonstrated a significant increase in PM_10_ concentrations compared to pre-earthquake levels in 2022, accompanied by a reported rise in respiratory diseases [2]. Although considerable time has elapsed since the earthquake, debris removal and reconstruction activities continue in certain areas, leading to regional variability in dust exposure that may persist for prolonged periods.

Despite the widespread and long-term nature of post-earthquake dust exposure, experimental evidence regarding the respiratory toxicity of specific building-derived dust components remains limited. In particular, the inhalation-related effects of hydrated cement dust (HCD), autoclaved aerated concrete dust (AACD) and DD on lung tissue have not been sufficiently characterized.

One of the most dangerous types of dust inhaled into the lungs is silica crystals, which have a chemical structure based on silicon. Prolonged inhalation of silica dust causes silicosis [3]. Spherical silica crystals with a dust diameter of less than 10 μm can reach the deep parts of the lungs after inhalation: the terminal bronchioles, alveolar ducts, and alveoli [4]. Initially, alveolar macrophages phagocytose silica dust [5] and produce large amounts of free oxygen and nitrogen radicals (e.g., superoxide, hydrogen peroxide, hydroxyl, nitric oxide) to destroy these phagocytosed silica particles. However, phagocytosed silica crystals cannot be eliminated in this way. The excessive production of free oxygen and nitrogen radicals in alveolar macrophages causes oxidative and nitrosative stress and oxidative/nitrosative tissue damage in alveolar macrophages and the lungs, leading to inflammation and apoptosis reactions [6,7].

The damage caused by increased oxidative stress in alveolar macrophages and the lungs leads to the release of numerous inflammatory factors [8]. Oxidative stress, oxidative damage, and inflammatory factors released into the surrounding tissue following excessive free radical production in lung tissue cause neutrophils to migrate to the damaged area of the lung and further complicate inflammation. The intensification of inflammation in the lung inhibits tissue repair and initiates fibrosis. The progression of fibrosis increases TGF-β1 release from macrophages and stimulates fibroblast activation [9]. Lung fibroblasts activated by TGF-β1 first transform into myofibroblasts, proliferate, migrate to the damaged tissue, and synthesize large amounts of collagen, thereby perpetuating fibrosis [10].

Numerous studies in the literature have examined the effects on the respiratory system of construction workers exposed to cement dust produced in a factory environment that has not yet hydrated [11,12,13,14,15]. However, studies on the toxic effects of HC particles, which are mixed with water and sand during construction, hydrate, harden into concrete, and then are reduced to powder form during demolition processes, are extremely limited. Similarly, experimental studies on the pulmonary toxicity of AACD and other DD are insufficiently represented in the literature. Dust exposure concentrations and exposure duration were determined based on previous particulate matter and dust exposure studies and to simulate the high-exposure conditions that may arise following the intense dust release after an earthquake. In a study conducted by M.W. Owonikoko et al. on rats, heavy metal accumulation in tissues and significant pathological changes in the lungs were reported after 14 days of cement dust exposure [16]. To our knowledge, this study is the first to evaluate the toxic effects of HCD, AACD, and DD on the lungs at the cellular and molecular levels following 14 days of exposure in rats. Therefore, the present study aims to investigate the respiratory toxicity of HCD, AACD, and DD using an experimental exposure model, focusing particularly on histopathological changes in lung tissue, in order to provide information on potential long-term respiratory health risks associated with post-earthquake air pollution. We believe that the findings of this study will contribute significantly to the existing literature and form a basis for future research in this field.

## 2. Materials and Methods

### 2.1. Preparation of HCD, AACD, and DD

To obtain hydrated cement (HC) in the laboratory, CEM I 42.5R cement, which is the most preferred type in concrete production, was used. In total, 1000 g of cement and 500 mL of tap water were mixed in a planetary mixer for 5 min to obtain fresh cement paste. The fresh cement paste was placed in a plastic container and tightly sealed. After being left at room temperature for 90 days, the sample was removed from the container, and HC was obtained. In the initial preparation stage, the hardened HC was coarsely crushed using a jaw crusher and sieved through a 1 mm mesh to obtain particles with a maximum size of 1 mm.

Autoclaved aerated concrete (AAC) was used as the main wall material in buildings that suffered severe damage in the earthquake. Therefore, this material also releases dust into the atmosphere during the demolition of these severely damaged buildings. The AACD used in this study was also collected from the surroundings of the demolished buildings and brought to the laboratory. Similarly, in the first processing step, the AAC material was coarsely crushed using a jaw crusher and passed through a 1 mm sieve to ensure a maximum particle size of 1 mm.

DD was collected during the controlled demolition of reinforced concrete structures that were severely damaged in the earthquake. Dust particles that settled on nylon sheets placed around the structure during demolition were collected and placed in closed containers, and subsequently passed through a 1 mm sieve to remove coarse particles.

All samples described above were placed in separate containers and dried in a laboratory oven at low temperature until a constant mass was obtained. The dried samples were then ground using a planetary ball mill operated at 300 rpm for 20 min.

Particle size analyses were performed on these ground samples. For particle size analysis, 2.5 g of each ground sample was weighed and dispersed in 50 mL of distilled water using a mechanical stirrer for 30 min to ensure a homogeneous suspension. The suspension was then sonicated in an ultrasonic bath for 10 min to further deagglomerate the particles. After sonication, the suspension was allowed to settle for 5 min, followed by filtration through blue band filter paper to remove coarse particles. The resulting filtrate was subsequently analyzed using a Malvern Zetasizer Nano ZS particle size analyzer (Malvern Panalytical Ltd., Malvern, UK). Each sample was measured three times to ensure repeatability. The particle size distributions of the HCD, AACD, and DD ranged from 1 to 10 μm. Particles within the 1–5 μm size range accounted for 82% of the HCD, 83% of the AACD, and 94% of the DD. These percentage distributions are consistent with previously reported values for micron-sized silica and silica-containing particles used in experimental pulmonary toxicity and fibrosis studies as well as inhalable particle formulations designed for pulmonary delivery [17,18,19,20,21,22,23].

### 2.2. Animals and Study Design

The protocol for this study was approved by the İnönü University Local Animal Experimentation Ethics Committee (Ethics Committee No: 2024/9-6). Twelve-week-old female Wistar albino rats (*n* = 48) weighing between 200 and 310 g were used in the study. The animals were obtained from the İnönü University Laboratory Animal Production and Research Center. During the experiment, they were housed in polycarbonate cages of appropriate size at 21 ± 2 °C and fed ad libitum with standard rodent chow and water. All animal care was performed under the supervision of a veterinarian in accordance with the Turkish Animal Protection Law. The National Institutes of Health’s Guide for the Care and Use of Laboratory Animals and the ARRIVE guidelines (Animals in Research: Reporting In Vivo Experiments) were followed throughout all experimental procedures [24]. Rats were randomly assigned to four groups with equal numbers of animals per group.

Group 1—Sham (Control) (*n* = 12): The group not exposed to dust, only administered sterile physiological saline.

Group 2—HCD (*n* = 12): This group was administered HCD suspended in 1 mL of sterile physiological saline, with 82% of the particles in the 1–5 μm size range.

Group 3—AACD (*n* = 12): This group was administered AACD suspended in 1 mL of sterile physiological saline, with 83% of the particles in the 1–5 μm size range.

Group 4—DD (*n* = 12): This group was administered DD suspended in 1 mL of sterile physiological saline, with 94% of the particles in the 1–5 μm size range.

HCD, AACD, and DD were suspended in sterile physiological saline at a concentration of 50 mg/mL and administered intranasally to rats at a volume of 0.1 mL (5 mg dust per rat) every 3 days for a total duration of 15 days, as described by Pu et al. [25]. Thus, all rats were exposed to HCD, AACD and DD for 2 weeks. At the end of the fifteenth day, all rats were euthanized by exsanguination under high-dose xylazine–ketamine anesthesia. Bronchoalveolar lavage (BAL) samples were collected from each animal, followed by thoracotomy for blood sampling. The lungs were removed in blocks, and the right lobe of the lung was fixed in 10% neutral formaldehyde solution and sent to the histology laboratory for histopathological evaluation.

### 2.3. Histological Analyses

In this study, histological and immunohistochemical stainings were performed using identical protocols, reagents, and incubation times. Sections from all experimental and control groups were stained within the same staining session. In addition, control group sections were included in each staining run to minimize technical variability. Immunohistochemical evaluation was based not only on visual staining patterns but also on semi-quantitative scoring of staining extent and intensity. All imaging and analyses were performed by two independent investigators using the same microscope, magnification, and image analysis settings.

At the end of the study, lung tissues were collected and the right lobe of the lung was fixed in 10% formaldehyde. Following fixation, the tissues were dehydrated and cleared, then embedded in paraffin blocks. Sections of 4 µm thickness were obtained from the paraffin blocks.

After deparaffinization and rehydration, the sections were stained with hematoxylin–eosin (H&E) to evaluate the structural components of the lung and with periodic acid–Schiff (PAS) to identify goblet cells. The stained sections were examined using an image analysis system.

Lung tissue damage was assessed and scored based on the following criteria: emphysema (enlargement of air sacs due to the loss of alveolar walls), infiltration of inflammatory cells (accumulation of macrophages and neutrophils in alveolar sacs, or perivascular or peribronchiolar regions), thickening of alveolar septa, and evidence of hemorrhage.

A semi-quantitative scoring scale was used for each parameter as follows: 0 = normal tissue; 1 = damage involving <25% of the total area; 2 = damage involving 25–50% of the area; 3 = damage involving >50% of the area [26].

Goblet cells in the airway epithelium were evaluated using a semi-quantitative scoring system based on the degree of PAS-positive staining: 0 = <5%, 1 = 5–25%, 2 = 26–50%, 3 = >50% of the epithelium showing PAS staining.

At least six randomly selected round or oval bronchioles were assessed; tangentially sectioned airways were excluded. An average score was calculated [27]. Analyses were performed at 20× objective magnification.

### 2.4. Bronchoalveolar Lavage Fluid (BALF) Analysis

Under anesthesia, a blunt needle connected to a syringe was inserted into the trachea to prepare for lung lavage. The lungs were lavaged four times with 3 mL of physiological saline via the tracheal cannula to collect the bronchoalveolar lavage fluid (BALF). The cell suspensions were concentrated by low-speed centrifugation, and the resulting cell pellet was resuspended. The obtained BALF samples were stained with Wright stain. Alveolar macrophages, epithelial cells, inflammatory cells, and erythrocytes were counted. Cell counting was performed under 40× objective magnification in 10 randomly selected microscopic fields, ensuring a minimum total of 200 cells. The percentage distribution of each cell type was then calculated [28].

### 2.5. Immunohistochemical Analysis

To identify macrophages in the alveolar lumen and interalveolar septa of the lung, CD68 was used; to evaluate fibrosis in the pulmonary parenchyma, TGF-β was applied.

For immunohistochemical evaluation, 4 µm thick tissue sections were mounted on poly-L-lysine-coated slides. Sections were passed through xylene and graded ethanol series for deparaffinization and rehydration. For antigen retrieval, the sections were placed in a sodium citrate buffer solution (pH 6.0), boiled in a pressure cooker for 20 min, and then cooled to room temperature.

To block endogenous peroxidase activity, the sections were incubated with 0.3% hydrogen peroxide solution for 15 min. Non-specific binding was prevented by incubating the sections with a protein blocking agent for 5 min. Then, the sections were incubated at room temperature for 60 min with the primary antibodies (CD68 1:100 and TGF-β 1:200; Santa Cruz Biotechnology, Inc., Heidelberg, Germany).

Following this, the sections were incubated with a biotinylated secondary antibody, followed by incubation with a streptavidin–peroxidase complex for 15 min. Immunoreactions were visualized using aminoethyl carbazole (AEC) as the chromogenic substrate. After staining, counterstaining was performed with hematoxylin. Finally, the slides were coverslipped using a water-based mounting medium and prepared for microscopic examination.

TGF-β and CD68 immunoreactivity was evaluated in 20 randomly selected fields per section based on two parameters: extent and intensity. The extent of staining was scored according to the percentage of positively stained area as follows: 0 = 0–25%, 1 = 26–50%, 3 = 51–75%, 4 = 76–100%. The intensity was graded as 0 = none, +1 = mild, +2 = moderate, +3 = strong staining. The total score for each field was calculated by multiplying the extent and intensity scores. All analyses were performed by two researchers using a Leica DFC280 light microscope and the Leica Q Win Image Analysis System (Leica Micros Imaging Solution Ltd., Cambridge, UK) [29].

### 2.6. Preparation of Lung Tissue Samples for Biochemical Analysis

On day 15 of the study, the right lobe of the lung of each rat was taken for biochemical analyses. The lung samples were homogenized using phosphate buffer saline to obtain the homogenate. The homogenate was centrifuged at 5000 rpm for 10 min to obtain the supernatant. The supernatants were used for biochemical analyses.

#### 2.6.1. Myeloperoxidase (MPO) Assay

The increase in MPO activity in tissues is considered the neutrophil accumulation index [30]. The MPO levels in the supernatant were measured using the method described by Bradley et al. [31]. The results were expressed as U/g protein.

#### 2.6.2. Hydroxyproline Assay

To analyze the level of collagen and lung fibrosis severity, lung hydroxyproline (Hyp) content was measured using the hydroxyproline assay method described by Bergman and Loxley [32]. The Hyp levels in lung tissue were detected at an absorbance of 550 nm. The results were expressed as μg Hyp/mg of lung weight.

#### 2.6.3. Western Blotting Analyses

Generally, inhaled silica-containing dusts cause lung damage by inducing oxidative stress, inflammation, apoptosis, and fibrosis in the lungs [33]. This experimental study aimed to analyze the levels of Akt, p-Akt, NF-KB, p-NF-KB, Bax, Bcl-xl, and caspase 9 in lung tissue of rats using Western blot to reveal all stages of the possible potential toxic effects of HCD, AACD and DD administered via inhalation.

The lung samples taken from the right lobe of the lung of each rat were lysed using radioimmunoprecipitation assay (RIPA) lysis buffer containing protease inhibitor cocktail (Bioshop Canada Inc.) and phenylmethanesulphonyl fluoride (PMSF), and the protein concentration was measured using a bicinchoninic acid (BCA) assay kit (Pierce ™ BCA Protein Assay Kit, Thermo Scientific, Rockford, IL, USA). The proteins were electrophoresed on sodium dodecyl sulphate–polyacrylamide gels and transferred to polyvinylidene fluoride (PVDF) membranes (GVS, Miami, FL, USA). After blocking with 5% milk for 1–2 h on a shaker, the membranes were incubated overnight at 4 °C with mouse anti-Akt1 (1/1000 and sc-5298 Santa Cruz Biotechnology, Inc., Dallas, TX, USA), mouse anti-phosphorylated-Akt1 (1/1000 and sc-293125 Santa Cruz Biotechnology, Inc., USA), rabbit anti-NF-KB p65 (1/1000 and #8242 Cell Signaling, Inc, Danvers, MA, USA), mouse anti-phosphorylated-NF-KB p65 (1/1000 and sc-136548 Santa Cruz Biotechnology, Inc., Dallas, TX, USA), mouse anti-Bax (1/1000 and sc-7480 Santa Cruz Biotechnology, Inc., Dallas, TX, USA), mouse anti-Bcl-xl (1/1000 and sc-8392 Santa Cruz Biotechnology, Inc., Dallas, TX, USA), mouse anti-caspase 9 (1/1000 and sc-56076 Santa Cruz Biotechnology, Inc., Dallas, TX, USA), and mouse anti-beta-actin (1/1000 and sc-47778 Santa Cruz Biotechnology, Inc., Dallas, TX, USA) antibodies. Then, the membranes were washed with Tris-buffered saline/Tween (TBST) four times for five minutes and incubated with HRP-conjugated secondary antibodies (anti-mouse 1/2000 7076S and anti-rabbit 1/2000 7074S Cell Signaling, Inc, Danvers, MA, USA) for 1–2 h at room temperature. Protein bands were detected using an enhanced chemiluminescence (ECL) kit (Pierce ™ ECL Western Blotting Substrate, Thermo Scientific, Rockford, IL, USA), and the intensities of protein bands were analyzed and quantified using ImageJ software version 1.53 to calculate relative expression by normalizing to beta-actin.

### 2.7. Statistical Analysis

Statistical analysis for histological and histochemical analyses was performed using the Windows-based IBM SPSS Statistics software (version 22.0; SPSS Inc., Chicago, IL, USA). The Shapiro–Wilk normality test was applied to evaluate the distribution characteristics of the dataset. Since the data did not show a normal distribution, the non-parametric Kruskal–Wallis test was used to examine differences between groups. For groups with significant differences according to this test, pairwise comparisons were conducted using the Bonferroni-corrected Mann–Whitney U test. A *p*-value of <0.05 was considered statistically significant. Data were presented as median (minimum–maximum) values. The non-parametric Kruskal–Wallis test was used for statistical analysis of biochemical data (Lung hydroxyproline and MPO) and Western blot data (Lung Akt1, p-Akt1, NF-κB, p-NF-κB, Bax, Bcl-xl, caspase 9), and the Conover post hoc test was used for intergroup comparison (1999, MedCalc 11.4). (*p* < 0.05) was considered statistically significant.

## 3. Results

### 3.1. XRD Analyses of HCD, AACD, and DD

In order to determine the hydration products in HCD, AACD, and DD, X-ray diffraction (XRD) analysis was performed using the dust samples administered to the mice. XRD analyses were performed using a Panalytical Empyrean diffractometer with Cu Kα radiation, and the diffraction patterns were recorded in the 2θ range of 5–90°.

**Figure 2:**
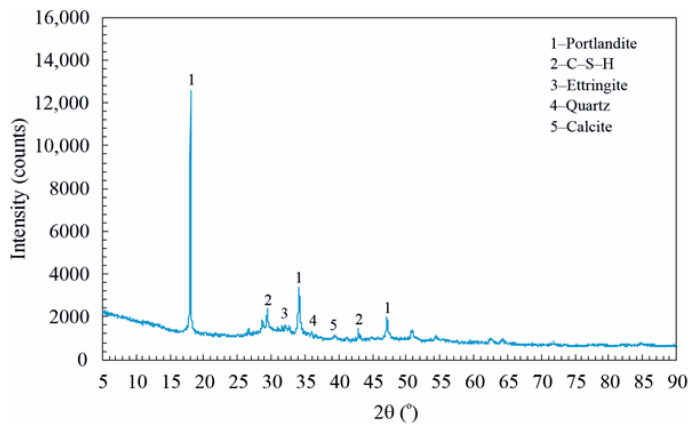
Hydration products of HCD.

Figure 2 shows the hydration products obtained by XRD analysis of HCD. It was observed that the hydration products in the HCD were portlandite (Ca(OH)_2_), C–S–H (3CaO·2SiO_2_·8H_2_O), ettringite (Ca_6_Al_2_(SO_4_)_3_(OH)_12_·26H_2_O), quartz (SiO_2_) and calcite (CaCO_3_) [34].

**Figure 3:**
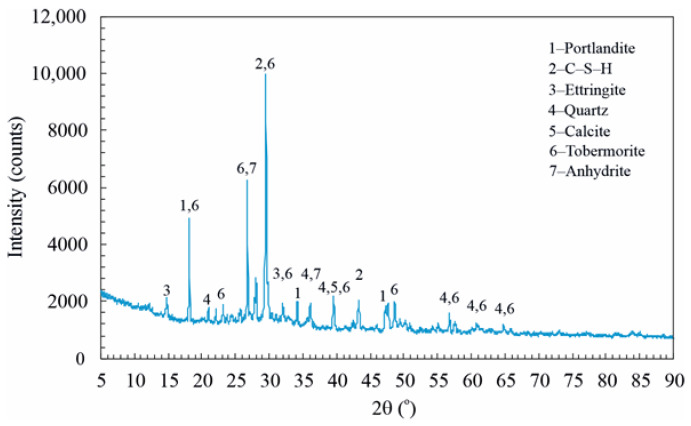
Hydration products of DD.

Figure 3 shows the XRD analysis of the DD. The hydration products in the demolition dust were observed to be portlandite, C–S–H, ettringite, quartz, calcite, tobermorite (Ca(OH)_2_·Ca_4_Si_6_O_16_·4H_2_O) and anhydrite (CaSO_4_).

**Figure 4:**
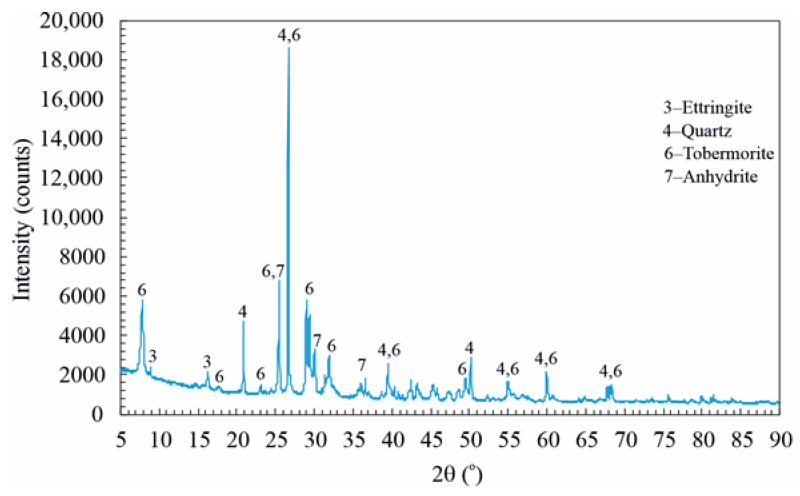
Hydration products of AACD.

Figure 4 shows the hydration products in AACD. The hydration products in AACD are portlandite, C–S–H, ettringite, quartz, calcite, tobermorite and anhydrite [35].

### 3.2. Effect of HCD, AACD and DD Administration on Akt1 and p-Akt1 Levels in the Lung

Akt1/beta-actin ratios of the control, AACD, HCD, and DD groups as the median (min–max) were found to be 0.007927 (0.005372–0.009074), 0.02245 (0.02175–0.04806), 0.03054 (0.02419–0.04806), and 0.01753 (0.01048–0.01889), respectively. p-Akt1/beta-actin ratios of the control, AACD, HCD, and DD groups as the median (min–max) were found to be 0.007160 (0.004923–0.007249), 0.01559 (0.01157–0.02344), 0.01124 (0.01091–0.01582), and 0.005088 (0.004524–0.008240), respectively.

**Figure 5:**
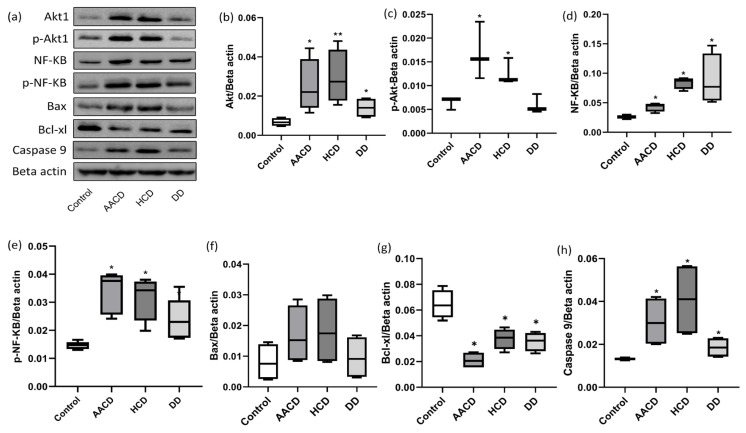
Rats treated with HCD, AACD, and DD administered via the respiratory route cause lung injury by activating the Akt1/NF-KB/caspase 9 pathway. (**a**) Western blot analysis of Akt1, p-Akt1, NF-KB, p-NF-KB, Bax, Bcl-xl, and caspase 9 proteins in the lung tissue of rats treated with AACD, HCD and DD. (**b**–**h**) The relative expression levels and statistical analysis of Akt, p-Akt1, NF-Kb, p-NF-KB, Bax, Bcl-xl, and caspase 9 proteins (*n* = 3 per group). Data are expressed as median ± min-max deviations; (*p* < 0.05) was considered statistically significant. * and ** mean statistically significant compared to the control group at (*p* < 0.05) and (*p* < 0.01), respectively. HCD: hydrated cement dust, AACD: autoclaved aerated concrete dust, DD: demolition dust.

Inhalation administration of HCD, AACD and DD to rats significantly increased Akt1 levels in the lungs compared to the control group (*p* < 0.05). Conversion of Akt1 to its active form, p-Akt1, was significantly increased only in the AACD and HCD group (*p* < 0.05). The p-Akt1 level in the DD group was not significant compared to the control group (*p* > 0.05) (Figure 5a–c).

### 3.3. Effect of HCD, AACD and DD Administration on NF-KB and p-NF-KB Levels in the Lung

The median (min–max) NF-KB/beta-actin ratios of the control, AACD, HCD, and DD groups were found to be 0.02637 (0.02635–0.02986), 0.04291 (0.03264–0.04757), 0.09035 (0.08423–0.09175), and 0.09352 (0.06067–0.1470), respectively. The median (min–max) P-NF-KB/beta-actin ratios of the control, AACD, HCD, and DD groups were found to be 0.01359 (0.01307–0.01662), 0.03929 (0.02707–0.03988), 0.03670 (0.02723–0.03802), and 0.02303 (0.01707–0.02584), respectively.

Administration of HCD, AACD and DD to rats significantly increased NF-KB and P-NF-KB levels in the lungs compared to the control group (*p* < 0.05) (Figure 5a,d,e). This result shows that the administration of HCD, AACD and DD to rats activates the NF-KB/p-NF-KB inflammation pathway and causes inflammation in the lung.

### 3.4. Effect of HCD, AACD and DD Administration on Bax and Bcl-xl Levels in the Lung

The median (min–max) Bax/beta-actin ratios of the control, AACD, HCD, and DD groups were found to be 0.01189 (0.002389–0.01455), 0.02073 (0.009690–0.02848), 0.02543 (0.008118–0.02984), and 0.01431 (0.003078–0.01678), respectively. The median (min–max) Bcl-xl/beta-actin ratios of the control, AACD, HCD, and DD groups were found to be 0.06206 (0.05184–0.07875), 0.01572 (0.01550–0.02724), 0.03952 (0.03758–0.04656), and 0.03924 (0.03340–0.04307), respectively.

Bax is a very important pro-apoptotic marker, while Bcl-xl is a very important anti-apoptotic protein. Administration of HCD, AACD and DD to rats significantly decreased Bcl-xl levels in the lungs compared to the control group (*p* < 0.05). Bax levels increased, but this increase was not statistically significant compared to the control group (*p* > 0.05) (Figure 5a,f,g). This result shows that administration of HCD, AACD and DD to rats activates the apoptotic pathway in the lung.

### 3.5. Effect of HCD, AACD and DD Administration on Caspase 9 Levels in the Lung

The median (min–max) caspase 9/beta-actin ratios of the control, AACD, HCD, and DD groups were found to be 0.01346 (0.01257–0.01392), 0.03898 (0.02013–0.04210), 0.05591 (0.02613–0.05645), and 0.02232 (0.01476–0.02302), respectively.

Apoptosis occurs primarily through caspase- and mitochondria-dependent pathways. In this regard, caspase-9 is the initiator of apoptosis. Administration of HCD, AACD and DD to rats significantly increased caspase-9 levels in the lungs compared to the control group (*p* < 0.05) (Figure 5a,h). This result indicates that administration of HCD, AACD and DD to rats initiates mitochondria-mediated apoptosis in the lung.

### 3.6. Effect of HCD, AACD and DD Administration on MPO Levels in the Lung

The median (min–max) lung MPO ratios of the control, AACD, HCD, and DD groups were found to be 579.9 (164.5–1214), 1610 (1265–2278), 1490 (862.2–2859), and 1312 (778.1–2461), respectively.

**Figure 6:**
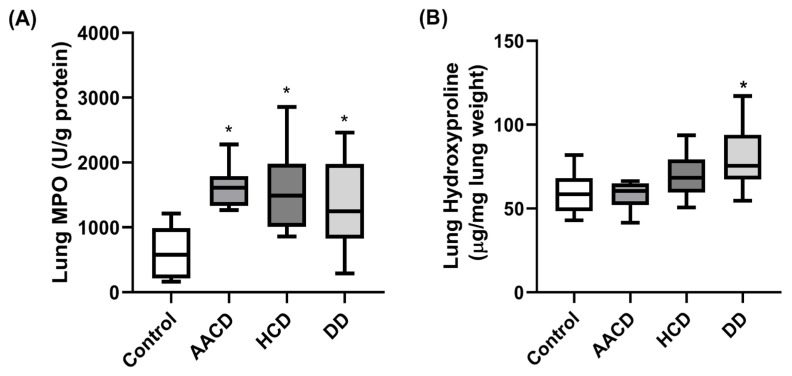
HCD, AACD and DD administered to rats via inhalation increased lung MPO levels statistically significantly (*p* < 0.05). Increased lung MPO levels indicated an increase in neutrophils in lung tissue of all groups. Only DD administration significantly increased lung hydroxyproline levels (*p* < 0.05) and caused lung fibrosis (histological image). (**A**) The levels of lung MPO in rats treated with HCD, AACD and DD (*n* = 10 per group). (**B**) The levels of lung hydroxyproline in rats treated with HCD, AACD and DD (*n* = 10 per group). Data are expressed as median ± min–max deviations; (*p* < 0.05) was considered statistically significant. * Statistically significant compared to the control group (*p* < 0.05). HCD: hydrated cement dust, AACD: autoclaved aerated concrete dust, DD: demolition dust.

The increase in MPO activity in damaged tissues is considered the neutrophil accumulation index. Administration of HCD, AACD and DD to rats significantly increased MPO levels in the lungs compared to the control group (*p* < 0.05) (Figure 6A). This result indicates the presence of a severe neutrophil sequestration in the lungs as a response to lung injury following administration of HCD, AACD and DD to rats.

### 3.7. Effect of HCD, AACD and DD Administration on Hydroxyproline Levels in the Lung

The median (min–max) lung hydroxyproline ratios of the control, AACD, HCD, and DD groups were found to be 58.53 (42.92–81.95), 60.48 (41.62–66.34), 68.29 (50.73–93.65), and 81.95 (68.94–117.1), respectively.

Hydroxyproline is an important parameter indicating the level of collagen synthesis and the severity of fibrosis. Among rats administered HCD, AACD and DD, only rats administered DD showed a significant increase in lung hydroxyproline levels compared to the control group (*p* < 0.05) (Figure 6B). The increase in lung hydroxyproline levels in the HCD and AACD groups was not statistically significant. This result suggests that DD administration to rats increases collagen synthesis and initiates fibrosis in the lung.

### 3.8. Effect of HCD, AACD and DD Administration on Histopathological Changes in the H&E- and PAS-Stained Lung Specimens

Emphysema, characterized by alveolar wall destruction, was found to be increased in all experimental groups compared to the control group. This increase was statistically significant in the HCD and AACD groups (*p* < 0.05), whereas the difference between the DD and control groups was not significant (*p* = 0.058). Although emphysema scores in the HCD and AACD groups were higher than in the DD group, the differences among the experimental groups were not statistically significant (*p* > 0.05). When comparing inflammation among groups, both the DD [2 (1.8–3)] and HCD [1.5 (1–3)] groups showed statistically significant increases compared to the control group [0.5 (0–1)] (*p* < 0.05), while the increase observed in the AACD group [1.3 (1–3)] was not significant. Although inflammation was more pronounced in the DD group, no significant difference was observed among the experimental groups (*p* > 0.05).

Regarding alveolar wall thickness scores, the highest values were detected in the HCD [2 (1.3–2.7)] and DD 1.9 (1.7–2.8)] groups, while the lowest score was observed in the AACD group [0.8 (0.2–1.3)]. A statistically significant increase (*p* < 0.05) was found in the HCD and DD groups compared to the control, whereas the AACD group showed no significant difference from the control (*p* > 0.05).

An increase in the density of magenta-stained goblet cells within the bronchial epithelium was observed in all experimental groups compared to the control. However, this increase was statistically significant only in the HCD group (*p* = 0.042), and no significant differences were found between the other groups (*p* > 0.05).

**Figure 7:**
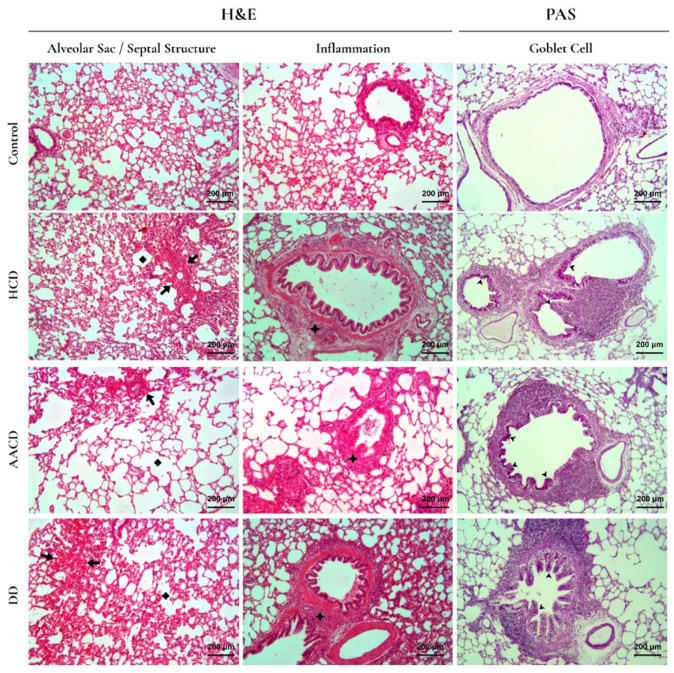
Increased alveolar wall thickness (arrow) in the HCD and DD groups, widespread emphysema (square) in the AACD group, and increased goblet cell density (arrowhead) in the HCD group are noteworthy (×10). HCD: hydrated cement dust, AACD: autoclaved aerated concrete dust, DD: demolition dust.

The histological evaluation scores are presented in Table 1, and comparisons of emphysema, inflammation, alveolar wall thickness, and goblet cell density among groups are shown in Figure 7.

### 3.9. Effect of HCD, AACD, and DD Administration on Immunohistochemical Changes in the Lung Specimens

**Figure 8:**
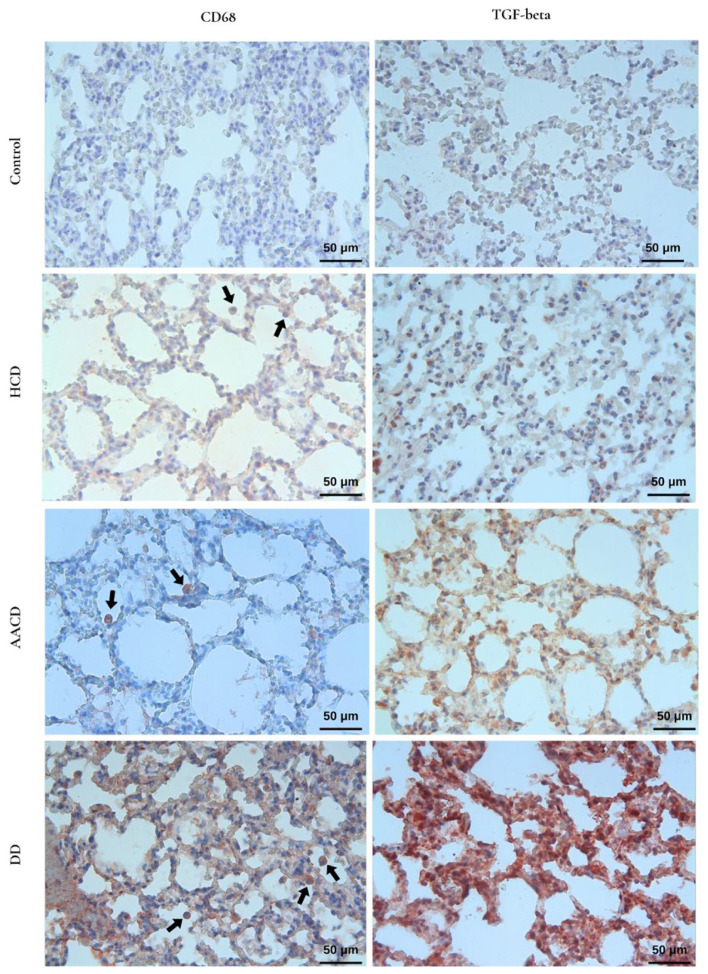
CD68(+) cells (arrow) and TGF-β immunoreactivity are most intense in the DD group. ×40. HCD: hydrated cement dust, AACD: autoclaved aerated concrete dust, DD: demolition dust.

CD68-positive macrophage cells, observed as brown-stained cells in the alveolar lumen and interstitial area, were increased in the experimental groups compared to the control group; however, this increase was statistically significant only in the DD group (*p* < 0.05). TGF-β staining, an indicator of fibrosis in the lung parenchyma, was prominent in all experimental groups. Compared to the control group, a statistically significant increase was detected only in the DD group (*p* = 0.002). When the experimental groups were compared among themselves, a significant difference was found only between the HCD and DD groups (*p* = 0.014) (Figure 8, Table 2).

### 3.10. Effect of HCD, AACD and DD Administration on Inflammatory Cell Count in the Bronchoalveolar Lavage Specimens

**Figure 9:**
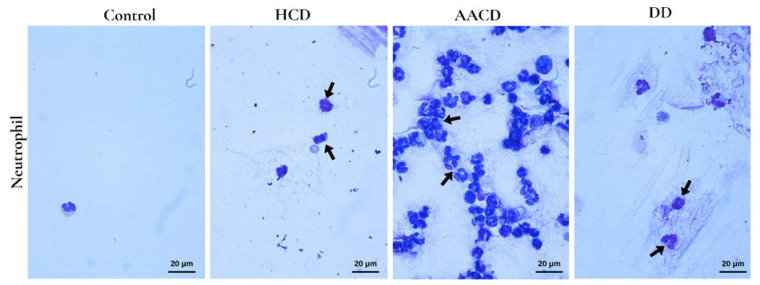
Polymorphonuclear neutrophils (arrow) observed in greater numbers in the AACD group compared to other groups. Wright stain, ×100. HCD: hydrated cement dust, AACD: autoclaved aerated concrete dust, DD: demolition dust.

To assess the lungs at the alveolar level, BALF cells (lymphocytes, neutrophils, and macrophages) were analyzed. Among the BALF cells, only the AACD group showed a statistically significant increase in neutrophil count compared to the control group (*p* = 0.006) (Figure 9).

Another cell type analyzed in the BALF, morphologically identified by a distinct nucleus and large vacuolated cytoplasm—macrophages—was increased in all experimental groups compared to the control group. However, no statistically significant differences were found among the groups (*p* > 0.05).

**Figure 10:**
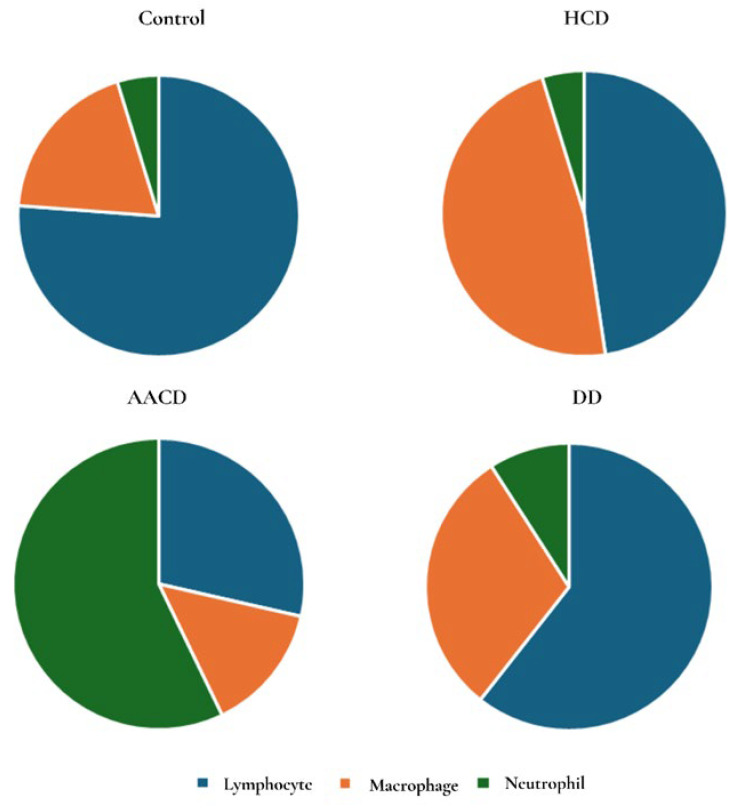
Distribution of lymphocyte, macrophage, and neutrophil counts in BAL fluid samples taken from all groups. HCD: hydrated cement dust, AACD: autoclaved aerated concrete dust, DD: demolition dust.

Additionally, lymphocytes, which were characterized by their round nuclei filling the cytoplasm, were found to be increased in all experimental groups compared to the control, but this increase was not statistically significant (*p* > 0.05) (Figure 10, Table 3).

## 4. Discussion

Our literature review on the subject reveals that numerous studies have been conducted examining the toxic effects of cement dust on human health [11,12,13,14,15]. However, no study has been found in the current literature that investigates the toxic effects of HCD, AACD and DD on living organisms. In this regard, our experimental study is the first to examine the damage and fibrosis occurring in the lungs at the molecular level by applying HCD, AACD and DD to rats via inhalation. HCD, AACD and DD were administered to the rats via inhalation five times during the 15-day study period. The lungs obtained from the rats were examined for histopathological changes using hematoxylin–eosin and immunohistochemical staining methods. In addition, to reveal molecular-level changes in the lungs, Akt-1, p-Akt1, NF-KB, p-NF-KB, Bcl-xl, Bax, caspase-9, myeloperoxidase, and hydroxyproline levels were analyzed using Western blot and ELISA methods. When our research results are evaluated together, it is revealed that the application of HCD, AACD and DD to rats causes inflammation, apoptosis, and damage in the lungs via the Akt1/NF-KB/Bcl-xl/caspase-9 signaling pathway, and that the application of DD additionally causes severe pulmonary fibrosis. We believe that the results of our study can make a significant contribution to the literature and serve as a basis for future research in this field.

The molecular mechanism(s) underlying the pathological changes and damage caused by the inhalation of silica-containing dust in the lungs are not fully understood. However, it has been suggested that lung damage caused by inhalation of silica dust may result from the combined effects of interconnected mechanisms such as the cytotoxic effect of silica on macrophages and alveolar epithelial cells, the production of excessive reactive oxygen and nitrogen species (ROS and RNS), the induction of inflammation, cytokine and chemokine production, cell apoptosis/pyroptosis, and pulmonary fibrosis [33,36,37,38,39,40]. The results of numerous studies indicate that the phosphoinositide 3-kinase (PI3K)/Akt (protein kinase B) signaling pathway may play a primary regulatory role in silica dust-induced lung damage [41,42,43].

Phosphoinositide 3-kinase (PI3K)/Akt (protein kinase B) signaling pathway is one of the intracellular signaling pathways involved in regulating various physiological processes, such as cell growth, proliferation, movement, metabolism, and survival [44]. Akt1 is widely found in tissue cells and contributes significantly to the mechanisms regulating cell proliferation and growth [45]. This supports the hypothesis that Akt1 may play a role in the pathogenesis of pulmonary fibrosis. Considering all the processes involved in the mechanism of damage caused by silica dust in the lungs, it can be readily stated that the PI3K-Akt signaling pathway and, possibly, its combined effect with oxidative stress may play a regulatory role in every stage of inflammation, apoptosis, and fibrosis involving alveolar macrophages, alveolar epithelial cells, and other cells of the lung. Research on this topic has revealed that the PI3K/Akt signaling pathway is activated in alveolar macrophages and alveolar epithelial cells in silica-induced lung damage [46].

Recent studies have reported that inhibition of the PI3K-Akt signaling pathway with antioxidant chemical agents prevents lung fibrosis [47,48]. Our findings indicate that HCD, AACD and DD administered to rats via inhalation likely reached the alveoli of the lungs due to their molecular size being much smaller than 10 µm and were taken up into the cells by alveolar macrophages and epithelial cells. Our Western blot analysis results on lung tissue revealed that HCD and AACD significantly increased levels of Akt and its active form, p-Akt, while DD had a lower level of increasing effect on Akt and p-Akt levels. These results indicate that HCD and AACD in particular strongly activate the Akt/p-Akt signaling pathway in the lungs.

NF-κB is considered the primary regulatory signaling cascade of inflammation due to its fundamental role in controlling every stage of the inflammatory response that occurs in the body [49,50]. Additionally, NF-κB has additional functions, such as cell survival, differentiation, proliferation, oxidative stress response, and tissue repair [51,52]. NF-κB can be activated by proinflammatory cytokines such as TNF-alpha and IL-1Beta, bacterial lipopolysaccharides, receptor ligands such as thrombin, and oxidative stress [53]. It has been reported that NF-κB is activated by silica in alveolar macrophages and epithelial cells and plays an important role in the initiation and progression of silica-induced pulmonary fibrosis [54,55,56,57]. Furthermore, Kane et al. reported that Akt may be an upstream activator of the NF-κB signaling cascade [58]. In their study on rats, Peng et al. reported that silica administration significantly increased p-Akt and p-NF-κB levels in the lungs, and that this increase was directly proportional to the severity of silica-induced pulmonary fibrosis [59]. Our Western blot results revealed that the application of HCD, AACD and DD to rats significantly increased levels of NF-κB and its active form, p-NF-κB. These results indicate that HCD, AACD and DD stimulate alveolar macrophages and epithelial cells, initiating pulmonary inflammation by increasing NF-κB and p-NF-κB expression in these cells. However, circulating neutrophils are recruited to the site of inflammation that chemotactically follow the inflammation signal, migrate to the inflammation site, become activated, and intensify inflammation [60,61]. Increased myeloperoxidase (MPO) activity in damaged tissues is accepted as an index of neutrophil accumulation [62]. In our current study, a significant increase in MPO activity was detected in the lungs of the groups exposed to HCD, AACD and DD compared to the control group. This increase in MPO activity indicates that the inflammation that begins in the lungs after the application of HCD, AACD and DD causes a large number of neutrophils to migrate and accumulate in the area, further intensifying pulmonary inflammation.

Apoptosis is a multisignal pathway process that plays a significant role in establishing and maintaining physiological homeostasis in tissues. It systematically eliminates cells in higher organisms that have sustained irreparable damage, lost metabolic control, and become dysfunctional. Apoptosis occurs primarily through two different signaling pathways: the intrinsic (mitochondrial) and extrinsic (death receptor) pathways. Initiator caspases (caspase-8 and -9) and executioner caspases (caspase-3, -6, and -7) are crucial apoptotic mediators that enable the execution of apoptotic signaling cascades [63]. The extrinsic pathway begins with the binding of ligands such as tumor necrosis factor (TNF) to the cell membrane death receptor [64], followed by the activation of caspase-8 and caspase-3, -6, and -7, leading to cell death [65]. Various stress signals, such as excessive reactive oxygen species (ROS) production, DNA damage, and endoplasmic reticulum stress, activate the intrinsic (mitochondrial) apoptotic pathway. This leads to increased permeability of the mitochondrial outer membrane, release of cytochrome c into the cytosol, and subsequent activation of caspase-9 and caspase-3, -6, and -7, leading to cell death [66,67].

The control and regulation of the intrinsic apoptotic pathway is carried out by proapoptotic proteins such as Bax and Bak and antiapoptotic proteins such as Bcl-2 and Bcl-xl in the Bcl-2 family. Bcl-2 and Bcl-xl control and regulate apoptosis prevention and cell survival, while Bax and Bak control and regulate apoptosis induction and cell death [68,69]. Our research results showed that, compared to the control group, Bcl-xl expression in the lungs decreased significantly in the groups treated with HCD, AACD and DD, while caspase-9 expression increased significantly. The increase in Bax expression was not statistically significant. The significant decrease in Bcl-xl levels and the significant increase in caspase-9 levels indicate that the intrinsic apoptotic pathway is activated, particularly in alveolar macrophages and epithelial cells, and that cell death has begun.

Alveolar macrophages are cells that play a crucial role in protecting the lungs from damage that may result from toxic particles reaching the alveoli. However, abnormal macrophage activation following the phagocytosis of toxic substances that cannot be easily eliminated, such as silica particles, by alveolar macrophages continues with the polarization of alveolar macrophages into M1/M2 macrophages and initiates pulmonary fibrosis, perpetuating the process [70]. In general, M1 macrophages stimulate inflammation by producing proinflammatory cytokines such as IL-1β, IL-6, and TNF-α, while M2 macrophages initiate fibrosis by producing TGF-β1, a key fibrotic cytokine in tissue repair [71]. Research using experimental animals and cell lines has confirmed that TGF-β1 is the primary fibrotic cytokine that initiates and perpetuates fibrosis [72,73,74]. It has been demonstrated that TGF-β1 released from M2 macrophages primarily activates fibroblasts and promotes their transformation into myofibroblasts [10]. Myofibroblasts have been reported to synthesize large amounts of collagen, thereby sustaining fibrosis [66]. Consistent with these results, the immunohistochemical analysis results of the current study indicate that the application of HCD, AACD and DD to rats increased the number of CD68-positive macrophages and TGF-β1 expression in the lungs, initiating fibrosis, but these increases are statistically significant only in the group treated with debris dust. BAL cell count results also partially support the increase in the number of CD68-positive macrophages.

When the data obtained in this study are evaluated together, it is revealed that the inhalation of AACD, HCD, and DD to rats for 15 days significantly disrupted the delicate balance between cell survival and death signals in lung tissue. Western blot analysis results show a significant but statistically insignificant suppression of the protective PI3K/Akt signaling pathway (a decrease in the p-Akt1/Akt1 ratio) in the AACD, HCD, and DD groups, and a statistically significant activation of the inflammatory NF-κB pathway (an increase in the p-NF-κB/NF-κB ratio) in the AACD and HCD groups. In the DD group, a significant suppression of the NF-κB signaling pathway (a decrease in the p-NF-κB/NF-κB ratio) occurred. As a result of the changes in these two signaling pathways, the level of anti-apoptotic Bcl-xl, a critical regulator of the mitochondrial apoptosis pathway, was significantly reduced, while the increase in pro-apoptotic Bax levels remained low, and the level of caspase-9, an effector of the intrinsic pathway, was significantly increased. These data point to a possible molecular mechanism suggesting that AACD, HCD, and DD exposure creates susceptibility to apoptosis by suppressing the cell’s endogenous protective mechanisms (Akt and Bcl-xl) rather than increasing pro-apoptotic signals. Namely, (1) Akt signaling positively regulates Bcl-xl expression by regulating transcription factors and other proteins to increase cell survival. In this study, the decrease in the p-Akt1/Akt1 ratio can be directly explained as the reason for the decrease in Bcl-xl levels. (2) Weakening of Akt signaling may have made the mitochondrial membrane more sensitive to pro-apoptotic stimuli (such as Bax) by reducing the production of anti-apoptotic Bcl-xl protein. (3) In the classical intrinsic apoptosis pathway, cell death signals are usually triggered by an increase in pro-apoptotic proteins such as Bax. However, our data suggest that in lung tissue cells exposed to AACD, HCD, and DD, apoptosis is initiated not by an increase in apoptosis-inducing factor (increase in Bax) but by a decrease in apoptosis-suppressing factor (decrease in Bcl-xl). This suggests that a decrease in anti-apoptotic protein levels such as Bcl-xl, rather than an increase in pro-apoptotic protein levels such as Bax, may be a sufficient and effective mechanism for regulating the apoptotic process.

Another important finding of this study is the significant increase in the p-NF-κB/NF-κB ratio in the AACD and HCD groups, and the significant decrease in the p-NF-κB/NF-κB ratio in the DD group. The significant increase in the p-NF-κB/NF-κB ratio in the AACD and HCD groups indicates that AACD and HCD exposure strongly activates the NF-κB signaling pathway by triggering an inflammatory response in lung tissue. Silica-induced toxicity in the lungs, occurring after the uptake of silica-containing particles by macrophages and epithelial cells, progresses as oxidative stress, inflammation, apoptosis, and fibrosis. The likely reason for the increased inflammation in the AACD and HCD groups due to the increased p-NF-κB/NF-κB ratio, and the decreased inflammation in the DD group due to the decreased p-NF-κB/NF-κB ratio, may be due to the very different characteristics and levels of the chemical compounds in AACD, HCD, and DD. Another possible reason is that exposure to DD can cause peracute toxicity in the lungs, with the inflammatory phase progressing very rapidly and triggering the fibrosis phase. The fact that TGF-β immunoreactivity and hydroxyproline levels, indicators of fibrosis, show a significant increase only in the DD group strongly supports this hypothesis.

## 5. Conclusions

Following acute inhalation of high concentrations of silica dust, oxidative stress, inflammation, apoptosis, and fibrosis occur at the cellular level in the lungs, resulting in lung damage. The current data suggest the inhalation of HCD, AACD and DD by rats caused inflammation, apoptosis, and damage in the lungs. However, histopathological, immunohistochemical, and biochemical analysis results show that DD application, in addition to causing inflammation, apoptosis, and damage in the lungs, significantly increases TGF-β1 expression, leading to a very pronounced level of fibrosis. When all the results obtained in the current study are evaluated together, it is concluded that the inhalation of HCD, AACD and DD in rats causes lung damage by modulating the Akt/NF-κB signaling cascade through the downregulation of the Bcl-xl anti-apoptotic protein and upregulation of Bax and caspase-9 pro-apoptotic proteins.

### Limitations

In this study, administering anesthesia to rats prior to each inhalation of dust may have caused stress in the animals. We believe that the different chemical contents administered to the rats in the study may also be a limitation for this study.

## Figures and Tables

**Table 1:** Histological evaluation.

Groups	Emphysema	Inflammation	Alveolar Wall Thickness	Goblet Cell
Control	0.5 (0.2–0.8)	0.5 (0–1)	0.3 (0–1.2)	0.5 (0.2–1.2)
HCD	1.8 (0.8–2.7) ^a^	1.5 (1–3) ^a^	2 (1.3–2.7) ^a,b^	1.3 (1.2–3) ^a^
AACD	1.8 (1–3) ^a^	1.3 (1–3)	0.8 (0.2–1.3)	1.5 (1.2–1.8)
DD	1.5 (1–2.3)	2 (1.8–3) ^a^	1.9 (1.7–2.8) ^a,c^	0.5 (0.2–2.2)

^a^ Statistically significant between the control and other groups (*p* < 0.05), ^b^ statistically significant between the HCD and AACD groups (*p* < 0.05), ^c^ statistically significant between the AACD and DD groups (*p* < 0.05). Table values are presented as median (min–max). HCD: hydrated cement dust, AACD: autoclaved aerated concrete dust, DD: demolition dust.

**Table 2:** Immunohistochemical findings.

Groups	CD68	TGF Beta
Control	2.6 (0.5–3.5)	2.8 (0.7–4.2)
HCD	3.2 (1–4.2)	3.2 (1.9–5.2)
AACD	3.5 (1.9–6.9)	3.6 (3.5–5.1)
DD	7 (3.9–9.6) ^a^	8.5 (5.7–9.5) ^a,b^

^a^ Statistically significant between the control and DD groups (*p* < 0.05). ^b^ Statistically significant between the HCD and DD groups (*p* < 0.05). Table values are presented as median (min–max). HCD: hydrated cement dust, AACD: autoclaved aerated concrete dust, DD: demolition dust.

**Table 3:** BAL evaluation.

Groups	Lymphocyte	Macrophage	Neutrophil
Control	0.08 (0–0.3)	0.02 (0–0.1)	0.005 (0)
HCD	0.1 (0.1–0.4)	0.1 (0–0.4)	0.01 (0–0.2)
AACD	0.2 (0–0.5)	0.1 (0–0.2)	0.4 (0.1–0.7) ^a^
DD	0.2 (0.1–0.6)	0.1 (0–0.3)	0.03 (0–0.3)

^a^ Statistically significant between the control and AACD groups (*p* < 0.05). Table values are presented as median (min–max). HCD: hydrated cement dust, AACD: autoclaved aerated concrete dust, DD: demolition dust.

## Data Availability

All data generated or analyzed during this study are included in this published article.

## Abbreviations

The following abbreviations are used in this manuscript:

HC	Hydrated cement
HCD	Hydrated cement dust
AAC	Autoclaved aerated concrete
AACD	Autoclaved aerated concrete dust
DD	Demolition dust
XRD	X-ray diffraction
FTIR	Fourier Transform Infrared Spectroscopy
CD68^+^	Cluster of differentiation 68
BAL	Bronchoalveolar lavage
BALF	Bronchoalveolar lavage fluid
PAS	Periodic acid–Schiff
TGF-β	Transforming growth factor beta
NF-κB	Nuclear factor kappa-B
Bcl-xl	B-cell lymphoma extra-large
Akt	Protein kinase B
p-Akt	Phosphorylated Akt
p-NF-κB	Phosphorylated nuclear factor kappa-B
AEC	Aminoethyl carbazole
Hyp	Hydroxyproline
MPO	Myeloperoxidase
Bax	Bcl-2-associated X protein
RIPA	Radioimmunoprecipitation assay
PMSF	Phenylmethanesulphonyl fluoride
BCA	Bicinchoninic acid
PVDF	Polyvinylidene fluoride
ECL	Chemiluminescence

## References

[1] Gandhi S.A., Min B., Fazio J.C., Johannson K.A., Steinmaus C., Reynolds C.J., Cummings K.J. (2024). The Impact of Occupational Exposures on the Risk of Idiopathic Pulmonary Fibrosis: A Systematic Review and Meta-Analysis. Ann. Am. Thorac. Soc..

[2] Bayram H., Rastgeldi Dogan T., Şahin Ü.A., Akdis C.A. (2023). Environmental and health hazards by massive earthquakes. Allergy.

[3] Wang M., Jiang C., Li S., Chen W., Zhang J., Zhao Y., Feng R., Han N., Shu G., Yin G. (2025). Submicron silica particles disrupt planarian homeostasis: Bridging bioaccumulation, oxidative stress, and growth-regeneration trade-offs. J. Mater. Chem. B.

[4] Adamcakova J., Mokra D. (2021). New Insights into Pathomechanisms and Treatment Possibilities for Lung Silicosis. Int. J. Mol. Sci..

[5] Shapouri-Moghaddam A., Mohammadian S., Vazini H., Taghadosi M., Esmaeili S.A., Mardani F., Seifi B., Mohammadi A., Afshari J.T., Sahebkar A. (2018). Macrophage plasticity, polarization, and function in health and disease. J. Cell Physiol..

[6] Gu K., Fu X., Tian H., Zhang Y., Li A., Wang Y., Wen Y., Gu W. (2020). TAZ promotes the proliferation and osteogenic differentiation of human periodontal ligament stem cells via the p-SMAD3. J. Cell Biochem..

[7] Jessop F., Hamilton R.F., Rhoderick J.F., Fletcher P., Holian A. (2017). Phagolysosome acidification is required for silica and engineered nanoparticle-induced lysosome membrane permeabilization and resultant NLRP3 inflammasome activity. Toxicol. Appl. Pharmacol..

[8] Tang Q., Xing C., Li M., Jia Q., Bo C., Zhang Z. (2022). Pirfenidone ameliorates pulmonary inflammation and fibrosis in a rat silicosis model by inhibiting macrophage polarization and JAK2/STAT3 signaling pathways. Ecotoxicol. Environ. Saf..

[9] Zhao Y., Hao C., Bao L., Wang D., Li Y., Qu Y., Ding M., Zhao A., Yao W. (2020). Silica particles disorganize the polarization of pulmonary macrophages in mice. Ecotoxicol. Environ. Saf..

[10] Ajayi I.O., Sisson T.H., Higgins P.D., Booth A.J., Sagana R.L., Huang S.K., White E.S., King J.E., Moore B.B., Horowitz J.C. (2013). X-linked inhibitor of apoptosis regulates lung fibroblast resistance to Fas-mediated apoptosis. Am. J. Respir. Cell Mol. Biol..

[11] Kim C.H., Park B., Baek M.S. (2025). The effect of long-term exposure to a mixture of air pollutants on chronic obstructive pulmonary disease. Ecotoxicol. Environ. Saf..

[12] Ramamoorthy T., Nath A., Singh S., Mathew S., Pant A., Sheela S., Kaur G., Sathishkumar K., Mathur P. (2024). Assessing the Global Impact of Ambient Air Pollution on Cancer Incidence and Mortality: A Comprehensive Meta-Analysis. JCO Glob. Oncol..

[13] de Souza Zorzenão P.C., dos Santos Silva J.C., Moreira C.A.B., Pinto V.M., de Souza Tadano Y., Yamamoto C.I., Godoi R.H.M. (2024). Impacts of PM2.5 exposure near cement facilities on human health and years of life lost: A case study in Brazil. J. Environ. Manag..

[14] Koh D.H., Kim T.W., Jang S.H., Ryu H.W. (2011). Cancer mortality and incidence in cement industry workers in Korea. Saf. Health Work.

[15] Eom S.Y., Cho E.B., Oh M.K., Kweon S.S., Nam H.S., Kim Y.D., Kim H. (2017). Increased incidence of respiratory tract cancers in people living near Portland cement plants in Korea. Int. Arch. Occup. Environ. Health.

[16] Owonikoko M.W., Emikpe B.O., Olaleye S.B. (2021). Standardized experimental model for cement dust exposure; tissue heavy metal bioaccumulation and pulmonary pathological changes in rats. Toxicol. Rep..

[17] Chen Y., Chen J., Dong J., Jin Y. (2004). Comparing study of the effect of nanosized silicon dioxide and microsized silicon dioxide on fibrogenesis in rats. Toxicol. Ind. Health.

[18] Yang T., Wang J., Pang Y., Dang X., Ren H., Liu Y., Chen M., Shang D. (2016). Emodin suppresses silica-induced lung fibrosis by promoting Sirt1 signaling via direct contact. Mol. Med. Rep..

[19] Zhang H., Sui J.N., Gao L., Guo J. (2007). Subcutaneous administration of infliximab-attenuated silica-induced lung fibrosis. Int. J. Occup. Med. Environ. Health.

[20] Xu X., Li Y., Niu Z., Xia J., Dai K., Wang C., Yao W., Guo Y., Deng X., He J. (2022). Inhibition of HIF-1α attenuates silica-induced pulmonary fibrosis. Int. J. Environ. Res. Public. Health.

[21] Yao J., Li Y., Meng F., Shen W., Wen H. (2023). Enhancement of suppression oxidative stress and inflammation of quercetin by nano-decoration for ameliorating silica-induced pulmonary fibrosis. Environ. Toxicol..

[22] Li B., Mu M., Sun Q., Cao H., Liu Q., Liu J., Zhang J., Xu K., Hu D., Tao X. (2021). A suitable silicosis mouse model was constructed by repeated inhalation of silica dust via nose. Toxicol. Lett..

[23] Xia J., Wang D., Guo W., Pei Y., Zhang L., Bao L., Li Y., Qu Y., Zhao Y., Hao C. (2024). Exposure to micron-grade silica particles triggers pulmonary fibrosis through cell-to-cell delivery of exosomal miR-107. Int. J. Biol. Macromol..

[24] Kilkenny C., Browne W., Cuthill I.C., Emerson M., Altman D.G. (2010). NC3Rs Reporting Guidelines Working Group. Animal research: Reporting in vivo experiments: The ARRIVE guidelines. Br. J. Pharmacol..

[25] Pu S., Yang Z., Zhang X., Li M., Han N., Yang X., He J., Yu G., Meng X., Jia Q. (2023). Fermented cordyceps powder alleviates silica-induced pulmonary inflammation and fibrosis in rats by regulating the Th immune response. Chin. Med..

[26] Rajizadeh M.A., Khoramipour K., Joukar S., Darvishzadeh-Mahani F., Iranpour M., Bejeshk M.A., Zaboli M.D. (2024). Lung molecular and histological changes in type 2 diabetic rats and its improvement by high-intensity interval training. BMC Pulm. Med..

[27] Huang X., Guan W., Xiang B., Wang W., Xie Y., Zheng J. (2022). MUC5B regulates goblet cell differentiation and reduces inflammation in a murine COPD model. Respir. Res..

[28] Kilic T., Parlakpinar H., Taslidere E., Yildiz S., Polat A., Vardi N., Colak C., Ermis H. (2015). Protective and therapeutic effect of apocynin on bleo-mycin-induced lung fibrosis in rats. Inflammation.

[29] Kose A., Parlakpinar H., Ozhan O., Ermis N., Yildiz A., Vardi N., Cigremis Y. (2020). Therapeutic effects of dexpanthenol on the cardiovas-cular and respiratory systems following cecal ligation and puncture-induced sepsis in rats. Biotech. Histochem..

[30] Brown K.E., Brunt E.M., Heinecke J.W. (2001). Immunohistochemical detection of myeloperoxidase and its oxidation products in Kupffer cells of human liver. Am. J. Pathol..

[31] Bradley P.P., Christensen R.D., Rothstein G. (1982). Cellular and extracellular myeloperoxidase in pyogenic inflammation. Blood.

[32] Bergman I., Loxley R. (1963). Two Improved and Simplified Methods for the Spectrophotometric Determination of Hydroxy-proline. Anal. Chem..

[33] Fubini B., Hubbard A. (2003). Reactive oxygen species (ROS) and reactive nitrogen species (RNS) generation by silica in inflammation and fibrosis. Free Radic. Biol. Med..

[34] Yaseen S.A., Yiseen G.A., Li Z. (2019). Elucidation of Calcite Structure of Calcium Carbonate Formation Based on Hydrated Cement Mixed with Graphene Oxide and Reduced Graphene Oxide. ACS Omega.

[35] Qu X., Zhao X. (2017). Previous and present investigations on the components, microstructure and main properties of autoclaved aerated concrete—A review. Constr. Build. Mater..

[36] Pollard K.M. (2016). Silica, Silicosis, and Autoimmunity. Front. Immunol..

[37] Hamilton R.F., Thakur S.A., Holian A. (2008). Silica binding and toxicity in alveolar macrophages. Free Radic. Biol. Med..

[38] Harijith A., Ebenezer D.L., Natarajan V. (2014). Reactive oxygen species at the crossroads of inflammasome and inflammation. Front. Physiol..

[39] Sayan M., Mossman B.T. (2016). The NLRP3 inflammasome in pathogenic particle and fibre-associated lung inflammation and diseases.. Part. Fibre Toxicol..

[40] Zhang L., He Y.L., Li Q.Z., Hao X.H., Zhang Z.F., Yuan J.X., Bai Y.P., Jun Y.L., Liu N., Chen G. (2014). N-acetylcysteine alleviated silica-induced lung fibrosis in rats by down-regulation of ROS and mitochondrial apoptosis signaling. Toxicol. Mech. Methods.

[41] Jia X., Li Z., Hu X., Wang T., Lian W., Sun W., Liu Y., Ni C. (2025). Homoharringtonine exerts anti-silicosis potential by inhibiting the CCR1 and PI3K/AKT signaling pathways in lung fibroblasts. J. Biomed. Res..

[42] Yang T., Pan Q., Yue R., Liu G., Zhou Y. (2024). Daphnetin alleviates silica-induced pulmonary inflammation and fibrosis by regulating the PI3K/AKT1 signaling pathway in mice. Int. Immunopharmacol..

[43] Qin Y., Wu Z., Zhang W., Zhong M., Liang Y., Wu J., Li Z., Nong Q., Huang Y., Sun H. (2025). Evodiamine attenuates silica-induced pulmonary fibrosis via PI3K/AKT pathway suppression: Integrated computational and experimental validation. Biochem. Biophys. Res. Commun..

[44] Yang J., Nie J., Ma X., Wei Y., Peng Y., Wei X. (2019). Targeting PI3K in cancer: Mechanisms and advances in clinical trials. Mol. Cancer.

[45] Larson-Casey J.L., Deshane J.S., Ryan A.J., Thannickal V.J., Carter A.B. (2016). Macrophage Akt1 Kinase-Mediated Mitophagy Modulates Apoptosis Resistance and Pulmonary Fibrosis. Immunity.

[46] Huo C., Jia Q., Jiao X., Jiang Q., Zeng X., Zhang J., Wang Y., Zhu Z., Tian L. (2025). Pulmonary microbiota affects silica-induced pulmonary fibrosis through activation of the PI3K/AKT-mediated senescence in alveolar epithelial cells. J. Hazard. Mater..

[47] Helal M.G., Said E. (2019). Carvedilol attenuates experimentally induced silicosis in rats via modulation of P-AKT/mTOR/TGFβ1 signaling. Int. Immunopharmacol..

[48] Ye Z., Niu Z., Li J., Li Z., Hu Y. (2024). Cardamonin inhibits silicosis development through the PI3K-AKT signaling pathway. Ecotoxicol. Environ. Saf..

[49] Ghosh S., Hayden M.S. (2008). New regulators of NF-kappaB in inflammation. Nat. Rev. Immunol..

[50] Zhang Q., Lenardo M.J., Baltimore D. (2017). 30 Years of NF-κB: A Blossoming of Relevance to Human Pathobiology. Cell.

[51] Hayden M.S., Ghosh S. (2008). Shared principles in NF-kappaB signaling. Cell.

[52] Hayden M.S., Ghosh S. (2012). NF-κB, the first quarter-century: Remarkable progress and outstanding questions. Genes. Dev..

[53] Millar M.W., Fazal F., Rahman A. (2022). Therapeutic Targeting of NF-κB in Acute Lung Injury: A Double-Edged Sword. Cells.

[54] Zhang J., Yang X., Yang Y., Xiong M., Li N., Ma L., Tian J., Yin H., Zhang L., Jin Y. (2022). NF-kB mediates silica-induced pulmonary inflammation by promoting the release of IL-1β in macrophages. Environ. Toxicol..

[55] Porter D.W., Ye J., Ma J., Barger M., Robinson V.A., Ramsey D., McLaurin J., Khan A., Landsittel D., Teass A. (2002). Time course of pulmonary response of rats to inhalation of crystalline silica: NF-kappa B activation, inflammation, cytokine production, and damage. Inhal. Toxicol..

[56] Di Giuseppe M., Gambelli F., Hoyle G.W., Lungarella G., Studer S.M., Richards T. (2009). Systemic inhibition of NF-kappaB activation protects from silicosis. PLoS ONE.

[57] Chen F., Shi X. (2002). NF kappaB, a pivotal transcription factor in silica induced diseases. Mol. Cell. Biochem..

[58] Kane L.P., Shapiro V.S., Stokoe D., Weiss A. (1999). Induction of NF-kappaB by the Akt/PKB kinase. Curr. Biol..

[59] Peng H.B., Wang R.X., Deng H.J., Wang Y.H., Tang J.D., Cao F.Y., Wang J.H. (2017). Protective effects of oleanolic acid on oxidative stress and the expression of cytokines and collagen by the AKT/NF κB pathway in silicotic rats. Mol. Med. Rep..

[60] Tsai Y.F., Chen C.Y., Yang S.C., Syu Y.T., Hwang T.L. (2023). Apremilast ameliorates acute respiratory distress syndrome by inhibiting neutrophil-induced oxidative stress. Biomed. J..

[61] Mortaz E., Alipoor S.D., Adcock I.M., Mumby S., Koenderman L. (2018). Update on Neutrophil Function in Severe Inflammation. Front. Immunol..

[62] Barone F.C., Hillegass L.M., Price W.J., White R.F., Lee E.V., Feuerstein G.Z. (1991). Polymorphonuclear leukocyte infiltration into cere-bral focal ischemic tissue: Myeloperoxidase activity assay and histologic verification. J. Neurosci. Res..

[63] Mustafa M., Ahmad R., Tantry I.Q., Ahmad W., Siddiqui S., Alam M., Abbas K., Hassan M.D.I., Habib S., Islam S. (2024). Apoptosis: A Comprehensive Overview of Signaling Pathways, Morphological Changes, and Physiological Significance and Therapeutic Implications. Cells.

[64] Green D.R. (2022). The Death Receptor Pathway of Apoptosis. Cold Spring Harb. Perspect. Biol..

[65] Nair P., Lu M., Petersen S., Ashkenazi A. (2014). Apoptosis initiation through the cell-extrinsic pathway. Methods Enzymol..

[66] Inoue S., Browne G., Melino G., Cohen G.M. (2009). Ordering of caspases in cells undergoing apoptosis by the intrinsic pathway. Cell Death Differ..

[67] Brentnall M., Rodriguez-Menocal L., De Guevara R.L., Cepero E., Boise L.H. (2013). Caspase-9, caspase-3 and caspase-7 have distinct roles during intrinsic apoptosis. BMC Cell Biol..

[68] Hardwick J.M., Soane L. (2013). Multiple functions of BCL-2 family proteins. Cold Spring Harb. Perspect. Biol..

[69] Kishore A., Petrek M. (2021). Roles of Macrophage Polarization and Macrophage-Derived miRNAs in Pulmonary Fibrosis. Front. Immunol..

[70] Zhang Z., Wu X., Han G., Shao B., Lin L., Jiang S. (2022). Altered M1/M2 polarization of alveolar macrophages is involved in the pathological responses of acute silicosis in rats in vivo. Toxicol. Ind. Health.

[71] Wolters P.J., Collard H.R., Jones K.D. (2014). Pathogenesis of idiopathic pulmonary fibrosis. Annu. Rev. Pathol..

[72] Vander Ark A., Cao J., Li X. (2018). TGF-β receptors: In and beyond TGF-β signaling. Cell Signal..

[73] Liu T.T., Sun H.F., Tang M.Z., Shen H.R., Shen Z., Han Y.X., Zhan Y., Jiang J.D. (2024). Bicyclol attenuates pulmonary fibrosis with silicosis via both canonical and non-canonical TGF-β1 signaling pathways. J. Transl. Med..

[74] Feng F., Li N., Cheng P., Zhang H., Wang H., Wang Y., Wang W. (2020). Tanshinone IIA attenuates silica-induced pulmonary fibrosis via inhibition of TGF-β1-Smad signaling pathway. Biomed. Pharmacother..

